# Evidence for the role of Rab11-positive recycling endosomes as intermediates in coronavirus egress from epithelial cells

**DOI:** 10.1007/s00418-022-02115-y

**Published:** 2022-05-23

**Authors:** Jaakko Saraste, Mary Enyioko, Hege Dale, Kristian Prydz, Carolyn Machamer

**Affiliations:** 1 Department of Biomedicine and Molecular Imaging Center (MIC), University of Bergen, Bergen, Norway; 2 Department of Biosciences, University of Oslo, Oslo, Norway; 3 Department of Cell Biology, Johns Hopkins University School of Medicine, Baltimore, MD USA

**Keywords:** Coronavirus (CoV), Pre-Golgi intermediate compartment (IC or ERGIC), Virus egress, Endocytic recycling compartment (ERC), Recycling endosome (RE), Rab11 GTPase

## Abstract

After their assembly by budding into the lumen of the intermediate compartment (IC) at the endoplasmic reticulum (ER)–Golgi interface, coronaviruses (CoVs) are released from their host cells following a pathway that remains poorly understood. The traditional view that CoV exit occurs via the constitutive secretory route has recently been questioned by studies suggesting that this process involves unconventional secretion. Here, using the avian infectious bronchitis virus (IBV) as a well-established model virus, we have applied confocal microscopy to investigate the pathway of CoV egress from epithelial Vero cells. We report a novel effect of IBV infection on cellular endomembranes, namely, the compaction of the pericentrosomal endocytic recycling compartment (ERC) defined by the GTPase Rab11, which coincides with the previously described Golgi fragmentation, as well as virus release. Despite Golgi disassembly, the IC elements containing the major IBV membrane protein (M)—which mostly associates with newly formed virus particles—maintain their close spatial connection with the Rab11-positive endocytic recycling system. Moreover, partial colocalization of the M protein with Rab11 was observed, whereas M displayed negligible overlap with LAMP-1, indicating that IBV egress does not occur via late endosomes or lysosomes. Synchronization of virus release using temperature-shift protocols was accompanied by increased colocalization of M and Rab11 in vesicular and vacuolar structures in the pericentrosomal region and at the cell periphery, most likely representing IBV-containing transport carriers. In conclusion, these results add CoVs to the growing list of viruses exploiting the endocytic recycling apparatus defined by Rab11 for their assembly and/or release.

**Supplementary Information:**

The online version contains supplementary material available at 10.1007/s00418-022-02115-y.

## Introduction

Instead of obtaining their membrane by budding at the cell surface—like, for example, influenza viruses—certain enveloped RNA and DNA viruses, such as bunya-, corona-, flavi-, toga- and herpesviruses, assemble by budding into the lumen of intracellular organelles, including the endoplasmic reticulum (ER), the intermediate compartment (IC), and the Golgi apparatus (Griffiths and Rottier 1992; Hernandez-Gonzalez et al. 2021; Saraste and Prydz 2021). Such an intracellular mode of multiplication requires that the progeny viruses are ultimately packaged into specialized transport carriers, which move towards the plasma membrane (PM) and undergo exocytosis, thereby resulting in virus release. Since these viruses form within compartments engaged in the secretory pathway, it has been generally assumed that their delivery to the extracellular space depends on constitutive secretion. This could also explain why the mechanisms of virus egress have received relatively little attention. However, the findings that these viruses typically cause an extensive reorganization of the Golgi apparatus have questioned the idea that their cellular exit involves conventional Golgi passage (Ruch and Machamer 2012; Saraste and Prydz 2021).

Coronaviruses (CoVs), a large family of positive-stranded RNA viruses, assemble by budding into the lumen of the IC at the ER–Golgi interface (Tooze et al. 1984; Klumperman et al. 1994; Saraste and Prydz 2021). On the basis of the detection of progeny CoVs at the dilated rims of Golgi cisternae, it was originally concluded that following their exit from the IC (“budding compartment”) the virus particles proceed across the Golgi stacks to reach the *trans*-Golgi network (TGN), where they are packaged into post-Golgi carriers for further delivery to the PM (Tooze et al. 1987; Salanueva et al. 1999; Machamer 2013). This concept on CoV release via constitutive secretion gained support from subsequent work, including recent studies of severe acute respiratory syndrome (SARS)-CoV-1 and SARS-CoV-2 (Siu et al. 2008; Bracquemond and Muriaux, 2021; Eymieux et al. 2021; Mendonça et al. 2021). However, evidence has also been presented that CoVs, such as SARS-CoV-2, can be released from their host cells in a Golgi-independent manner by employing lysosomal secretion (Ghosh et al. 2020). Moreover, we recently proposed that the cellular exit of CoVs could involve an unconventional secretory pathway based on a direct functional connection between the IC and the endocytic recycling system (Saraste and Prydz 2021). In fact, the endocytic recycling apparatus defined by the GTPase Rab11 is known to play an important role in the assembly and/or release of a number of enveloped viruses budding either intracellularly or at the cell surface (Bruce et al. 2012; Vale-Costa and Amorim 2016; Lucin et al. 2018), encouraging examination of its possible participation in the late stages of the CoV life cycle.

Using high-resolution confocal microscopy (CM) to investigate epithelial green monkey kidney (Vero) cells infected with avian bronchitis virus (IBV)—a γ-CoV that shares many features with β-CoVs such as SARS-CoV-2—we describe a novel effect of IBV infection on the cellular endomembrane system. Interestingly, the observed reorganization of the endocytic recycling apparatus defined by the GTPase Rab11—based on the accumulation of recycling endosomes (REs) next to the centrosome—appears to be intimately linked to IBV-induced Golgi fragmentation and virus release. Accordingly, we provide evidence supporting the function of Rab11-positive REs as transport carriers during the cellular exit of the virus.

## Materials and methods

### Cell culture

Green monkey kidney epithelial (Vero) cells, originally obtained from the European Collection of Authenticated Cell Cultures (ECACC; 84,113,001), were kindly provided by Drs. Yuta Ishizuka and Clive Bramham. The cells were grown at 37 °C in 5% CO_2_ atmosphere in Dulbecco’s Minimum Essential Medium (DMEM) with low glucose (1 g/L) and supplemented with 10% heat-inactivated fetal calf serum (FCS), 2 mM l-glutamine, 50 units/ml penicillin, and 50 µg/ml streptomycin. For microscopy, the cells were plated on 18-mm-diameter glass coverslips on six-well plates and grown for 2 days to reach a confluency of 60–70%. For the preparation of virus stocks, determination of virus release, and plaque titration of medium samples, the cells were grown on 10-cm-diameter dishes or six-well plates for 2 days until they reached 80–90% confluency.

### Virus infection

Avian infectious bronchitis virus (IBV) is a γ-CoV, which causes respiratory and gastrointestinal disease in birds but is harmless to humans, allowing the experiments to be carried out in a BSL-2 facility. For the preparation of virus stocks, Vero cells grown in two 10-cm-diameter culture dishes were washed with serum-free DMEM and infected with IBV diluted in the same medium to give a low multiplicity of infection (MOI) of 0.1 plaque-forming units (PFU)/cell. After 60 min incubation in a CO_2_ incubator at 37 °C, the virus was removed and 6 ml DMEM containing 2% FCS was added to each plate. Incubation of the infected cultures was continued for up to 20 h until the formation of syncytia was observed by phase-contrast microscopy. At harvest, the dishes were covered tightly with Parafilm and subjected to three rounds of freezing (−80 °C freezer) and thawing (37 °C warm plate) to also collect the intracellular progeny viruses. After careful mixing, the virus-containing homogenates were transferred into 15 ml tubes, vortexed extensively, and centrifuged for 15 min at 2000 rpm. The supernatants were divided into 250 µl and 500 µl aliquots and stored in a −80 °C freezer. The virus stocks reached titers of up to 1 × 10^7^ PFU/ml.

Initially, the number of infected cells on the glass coverslips prepared for microscopy was low (about 20%) and variable between experiments. Therefore, to increase the efficiency of infection, two modifications were introduced. First, addition of the virus in a small volume (50 µl) just on top of the coverslips allowed long-term use of the same virus stock at a relatively high MOI (up to 2 PFU/cell). Second, virus adsorption was carried out at low temperature by keeping the coverslip-containing six-well plates for 60 min on ice, prior to their placement in the 37 °C incubator for another 60 min. In combination, these modifications gave a > 2.5-fold increase in the efficiency of infection—that is, infection of more than 50% of the cells.

### Plaque assay

**Fig. 1:**
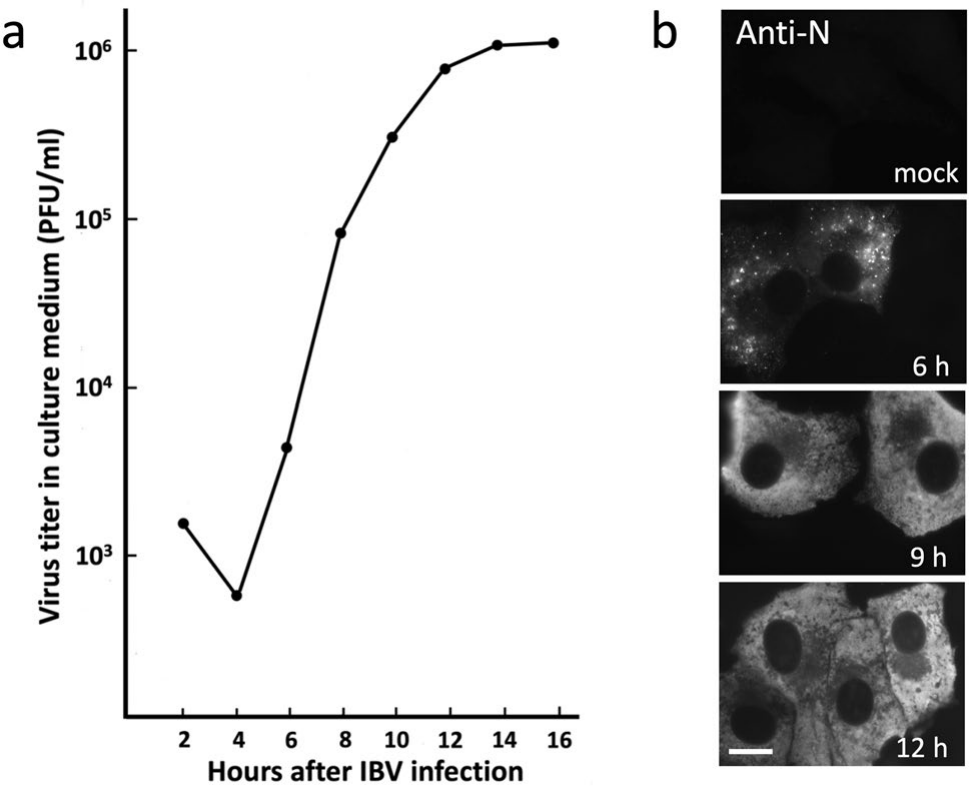
**a** Kinetics of IBV release from Vero cells. Medium samples from the virus-infected cell cultures incubated at 37 °C were harvested at 2 h intervals and subjected to plaque titration to determine the time course of release of infectious virus. Note that the release of progeny virus starts between 5 and 6 h post-infection (hpi), while the earlier measurements score residual cell-attached virus from the original inoculum. **b** Immunofluorescence microscopic localization of the IBV N protein in mock-infected Vero cells and at different times after infection. At 6 hpi the anti-N antibodies give a weak punctate pattern, while at 9 and 12 hpi the infected cells typically display strong diffuse staining due to high N expression. Bar, 10 μm

Cells on six-well plates were infected at a MOI of 1 PFU/cell, and medium samples were recovered at the indicated timepoints following incubation of the cultures at 37 °C or 31 °C (Figs. 1 and 9). Tenfold serial dilutions (10^–1^–10^–6^) of the samples were prepared, and 200 µl aliquots were used to infect cells grown in six-well plates. At the end of infection, melted 1.8% agarose solution kept at 55 °C was combined with an equal volume of prewarmed 2× DMEM supplemented with 4% FCS and allowed to cool to 37 °C. Following removal of the virus, 2 ml of the mix was added into each well of the plate until it solidified, whereafter the culture plates were placed in a 37 °C CO_2_ incubator for 2 days to allow plaque formation. After keeping the plates for 30 min in the refrigerator, the solid agarose overlay was removed with a spatula and the cells in each well were stained for 10 min with 1 ml of 0.05% crystal violet in 20% EtOH. The staining solution was removed, and the wells were rinsed with ddH_2_O and allowed to dry. The virus titers were determined from wells containing 10–100 plaques.

### Antibodies

The preparation of affinity-purified rabbit antibodies against the IBV M-protein has been described previously (Machamer and Rose 1987). The rabbit antibody against the IBV N protein was a kind gift from Dr. Ellen Collisson (University of Health Sciences, Pomona, CA, USA). The production and affinity purification of rabbit antibodies against Rab1 has been described previously (Saraste et al. 1995). The monoclonal antibody against Rab1B (1E7) was kindly provided by Dr. Angelica Barnekow (University of Münster, Germany). The commercial antibodies were purchased from the following sources: monoclonal mouse anti-Rab11 (clone 47) and anti-GM130 (clone 35) from BD Transduction Laboratories; the polyclonal (rabbit) and monoclonal (mouse) antibodies against p58/ERGIC-53 from Sigma (E1031) and Alexis (G1/93), respectively; mouse monoclonal anti-LAMP-1 (H4A3) from Abcam; polyclonal rabbit anti-GalNT2 (B75232) from Sigma; and mouse monoclonal antibody against human transferrin receptor (H68.4) from Invitrogen. The secondary Alexa 488- or Alexa 594-coupled goat anti-rabbit or anti-mouse F(ab)_2_-fragments were purchased from Jackson ImmunoResearch Laboratories.

### Immunofluorescence staining

At harvest, the cells grown on glass coverslips were fixed for 60 min with 3% paraformaldehyde in 0.1 M phosphate buffer, pH 7.2. The detailed staining protocol, involving permeabilization of the cells with 0.2% saponin, has been described previously (Sannerud et al. 2008). For staining with the monoclonal anti-Rab11 antibodies, the fixed cells were first treated for 5 min with 6 M guanidine–HCl in 50 mM Tris buffer, pH 7.5, to expose the antigenic sites, followed by extensive washing with PBS containing 0.2% BSA, permeabilization, and staining with the primary and secondary antibodies. Incubations with the primary antibodies were usually carried out overnight in a humid chamber. After completion of the staining, the cells were mounted on EtOH-washed objective slides in 10 µl of Vectashield Mounting Medium containing DAPI (Vector Laboratories).

### Microscopy and quantification

The antibody-stained cells were usually first examined in a Zeiss Axiovert 200 M inverted microscope equipped with long-working-distance Plan-NEOFLUAR 40× (dry) and 100× (oil immersion) objectives, phase-contrast capability, AxioCam HRm camera and fluorescence filters appropriate for the two fluorophores (Alexa 488 and Alexa 596), and DAPI. Confocal microscopy (CM) on optimal specimens was performed on a Leica TCS SP8 system (Leica Microsystems, Germany), and individual optical sections or *z*-stacks (step size 0.3 µm) were acquired with a 100× NA1.4 HC PL APO STED White objective. The images were processed with the integrated adaptive deconvolution module Lightning and presented as single sections or maximum-intensity projections.

The extent of Golgi fragmentation and ERC compaction in the course of IBV infection—using ERGIC-53 or GM130 and Rab11 as the markers for *cis*-Golgi and the ERC, respectively—were determined from 100–200 cells per timepoint by employing double-staining for the N protein to identify the infected cells. Colocalization analysis of images double-stained for the IBV M protein and Rab11 or LAMP-1 was carried out as described by Dunn et al. (2011) by employing the Imaris software (Oxford Instruments) to determine the scatter plots and Pearson’s correlation coefficients (PPCs; Supplementary Fig. S1).

## Results

### Characteristics of IBV replication in Vero cells

To define certain basic parameters of IBV infection in Vero cells, we first examined the kinetics of virus release. Samples of the growth medium were recovered every 2 h until 16 h post-infection (hpi), and the amounts of infectious virus released from the cells were measured by plaque titration. The first progeny viruses started to appear in the medium at 5–6 hpi, whereafter an exponential increase in virus release took place until 12 hpi, finally reaching a plateau at 12–14 hpi. On the basis of the growth curve (Fig. 1a)—to avoid cellular changes that lead to cessation in the release of infectious virus late during infection—the experiments described below focused on the active phase of virus replication and release between 6 and 12 hpi.

**Fig. 2:**
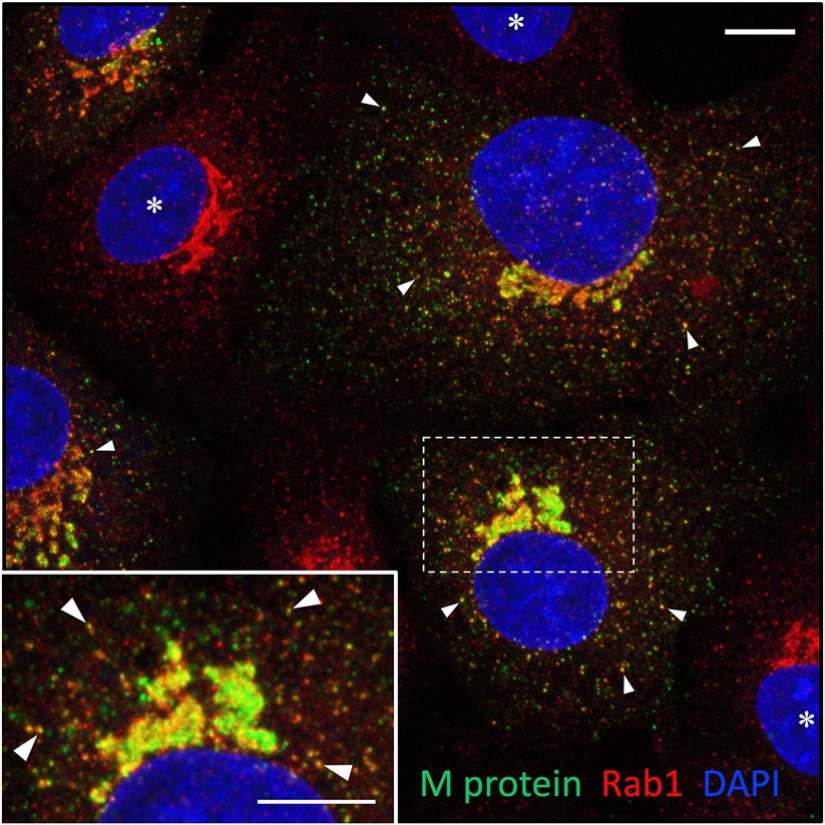
The IBV M-protein colocalizes extensively with IC marker Rab1. The IBV-infected cells were fixed at 8 hpi and double-stained for CM using antibodies against Rab1 and the M protein, which predominantly associates with intralumenal virus particles. Note the variable expression of the M protein in different virus-infected cells. The inset highlights the Golgi region of a cell with strong M expression, leading to the development of membrane domains that evidently contain progeny virus, but are devoid of Rab1. Peripheral IC elements positive for both M and Rab1 are indicated by arrowheads, while uninfected cells are denoted by asterisks. The nuclei were visualized by DAPI staining. Bars, 5 μm

Next, to reveal the time course of synthesis of the structural proteins of IBV, cells fixed at various times of infection were stained with antibodies against the cytoplasmic nucleocapsid (N) protein. Punctate staining for the N protein was observed at 6 hpi, whereas at later timepoints (9 and 12 hpi) an increasing number of the cells displayed strong diffuse staining throughout the cytoplasm (Fig. 1b), similarly as reported for Vero cells infected with SARS-CoV-2 (Scherer et al. 2022). Using antibody staining, we also examined expression of the M protein, the major membrane protein of the viral envelope, which plays an important role in the budding process (Vennema et al. 1996; de Haan et al. 1998; Bracquemond and Muriaux 2021). While only negligible perinuclear Golgi-like staining for the M protein was detected at 6 hpi (data not shown), strong M-specific fluorescence was seen in cells at 8 hpi (Fig. 2; see also Scherer et al. 2022).

Therefore, to visualize the sites of virus assembly and budding, we compared distributions of the M protein and the GTPase Rab1 in cells fixed at 8 hpi (Fig. 2). Namely, previous immunoelectron microscopy (IEM) studies applying different antibodies have shown that the M protein predominantly associates with CoV particles residing in the lumen of IC or Golgi elements, or inside virus-containing transport carriers (Ulasli et al. 2012; Ghosh et al. 2020). Rab1 is a well-characterized component of the IC transport machinery (Saraste 2016) and has been employed as a specific IC marker in IEM to identify the CoV “budding compartment” (Krijnse-Locker et al. 1994). As expected, CM showed extensive colocalization of Rab1 and the M protein in the juxtanuclear Golgi region and in punctate structures scattered throughout the cytoplasm (Fig. 2). The majority (63.4%) of the Rab1-positive IC elements also contained the M protein, most likely corresponding to pre-Golgi sites of IBV budding or trafficking. About one-third (31%) of the M-protein-positive structures lacked detectable Rab1, possibly representing virus-containing exocytic carriers.

**Fig. 3:**
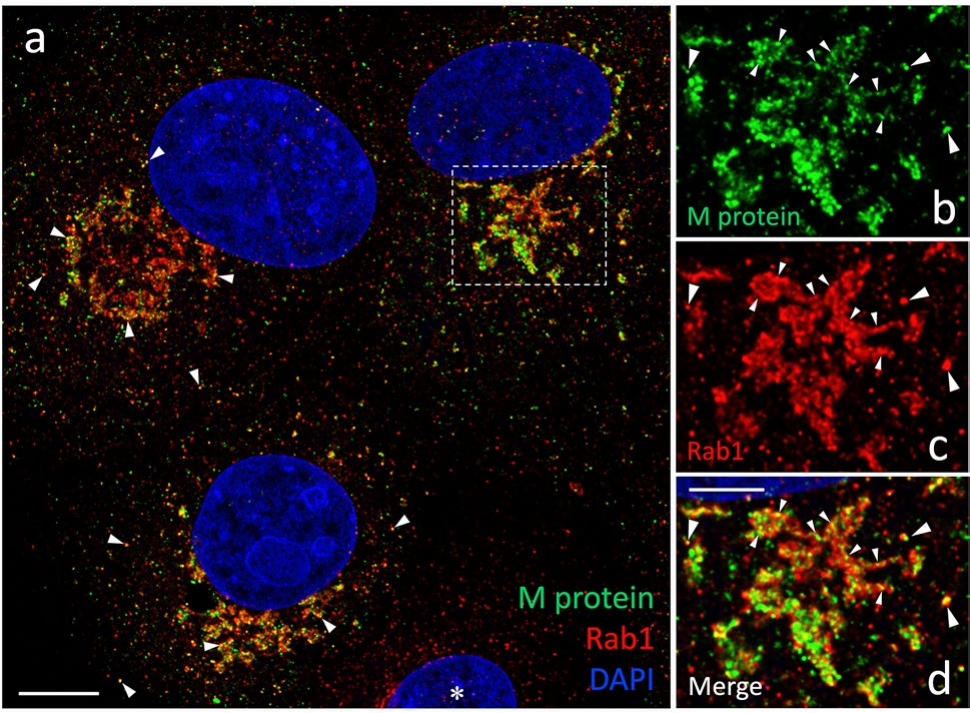
Comparing the localizations of the IBV M protein and Rab1 at higher resolution. The cells fixed at 8 hpi were stained with antibodies and DAPI as in Fig. 2 and subjected to image deconvolution. The M protein and Rab1 show considerable overlap in peripheral and central IC elements, as demonstrated by the merged image in **a** (arrowheads). However, the individual magnified images in **b**–**d**—corresponding to the Golgi area of a cell with higher M expression indicated in **a**—reveal partly non-overlapping distributions of the two proteins both in larger IC structures (large arrowheads) as well as along the Golgi ribbon that displays partial fragmentation (small arrowheads). Bars, 5 μm (**a**) and 2.5 μm (**b–d)**

The M protein and Rab1 displayed largely overlapping distributions in the Golgi region of cells at an early stage of infection, expressing moderate amounts of the viral membrane protein (Fig. 2). Instead, in cells expressing higher amounts of the M protein—which also show signs of Golgi fragmentation (see below)—the protein was found to increasingly segregate into separate membrane domains lacking detectable Rab1 (Fig. 2, inset). Such domains are likely to correspond to dilated IC- or Golgi-derived structures containing large amounts of progeny virus, which have previously been demonstrated by EM (Tooze et al. 1987; Ulasli et al. 2012; Eymieux et al. 2021). To compare the cellular localizations of M and Rab1 at higher resolution, we applied image deconvolution. While the two proteins maintained considerable overlap after image processing (Fig. 3a), their partial enrichment in distinct membrane domains—both within large IC elements as well as along the fragmented Golgi ribbon—became more obvious (Fig. 3b–d). Some of the large M-positive puncta in the Golgi region devoid of Rab1 may correspond to exocytic transport carriers containing progeny virus (see below).

### IBV causes compaction of the Rab11-positive ERC

Recycling endosomes (REs)—defined by the GTPase Rab11, a master regulator of endocytic membrane recycling (Ullrich et al. 1996; Ren et al. 1998), or the transferrin receptor (TfR)—constitute a dynamic membrane system. Based on their motor-dependent movements along cytoskeletal tracks, either actin filaments or microtubules (MTs), they can assume a more widespread distribution in certain cells, or accumulate at the cell center in others, establishing the pericentrosomal endocytic recycling compartment (ERC) (Maxfield and McGraw 2004; Misaki et al. 2010; Naslavsky and Caplan 2018). Moreover, a subpopulation of REs has recently been shown to closely associate with the *trans*-side of the Golgi stacks (Fujii et al. 2020).

**Fig. 4:**
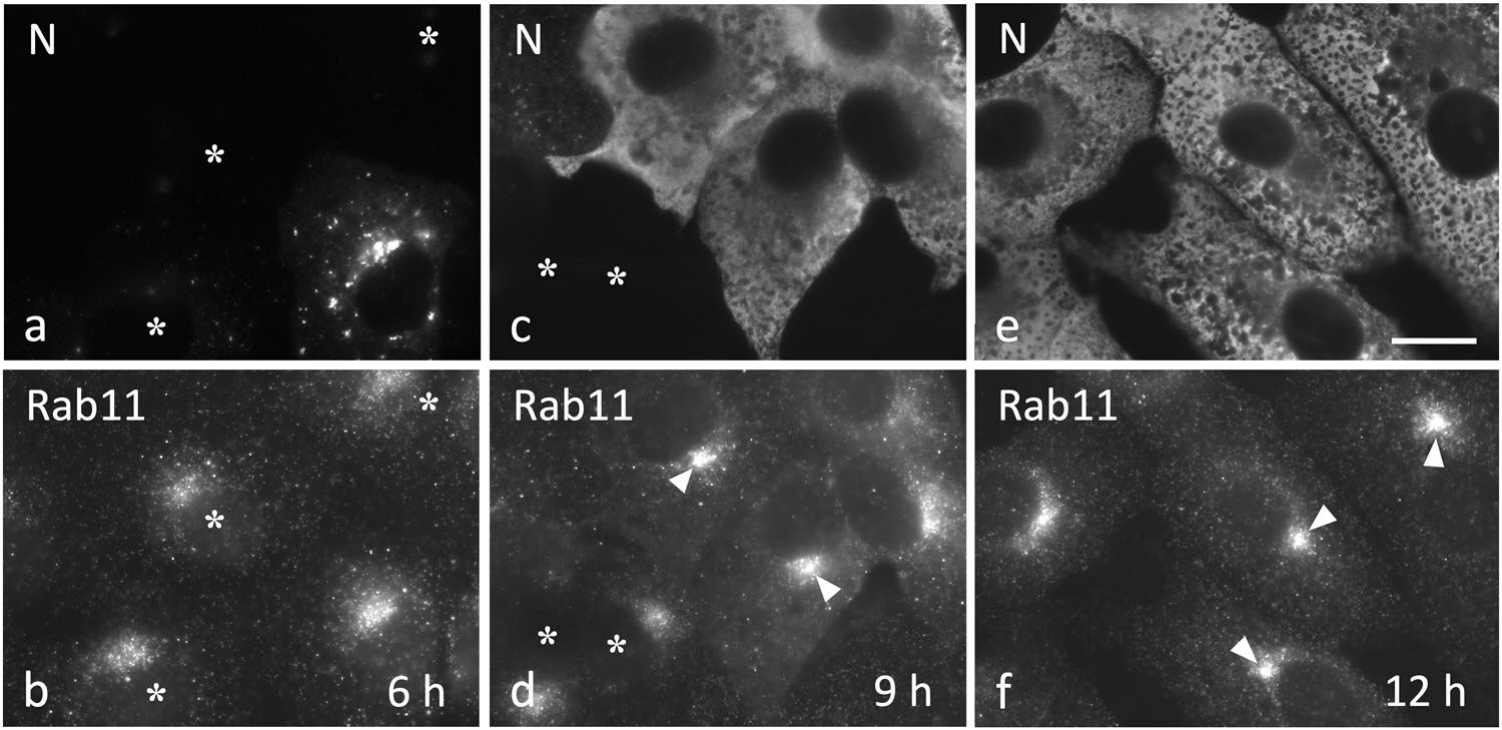
IBV infection of Vero cells results in ERC compaction. The cells fixed at 6, 9, or 12 hpi were double-stained for immunofluorescence microscopy with antibodies against Rab11 and the viral N protein—to localize REs and identify infected and uninfected (asterisks) cells, respectively. Note the gradual compaction of the Rab11-positive pericentrosomal ERC (arrowheads) in the course of the infection and simultaneous reduction of the peripheral Rab11 signal. Bar, 10 μm

**Fig. 5:**
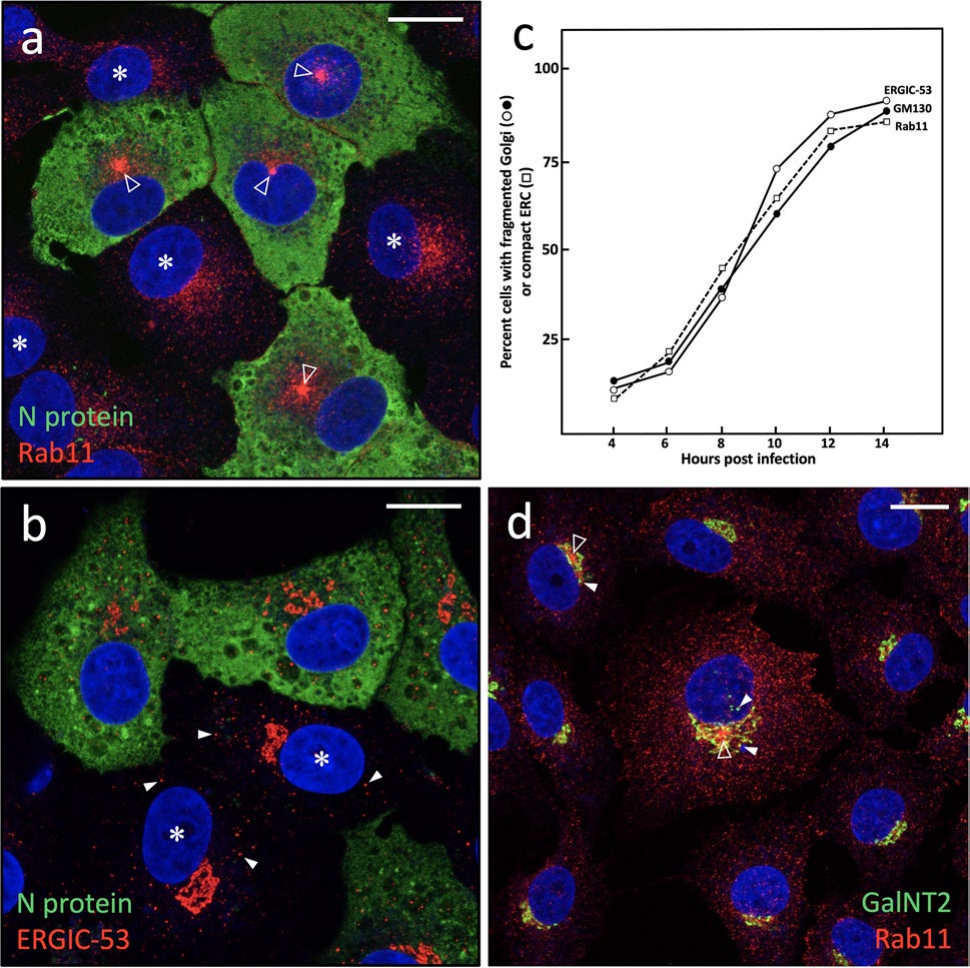
IBV-induced ERC compaction and Golgi fragmentation follow similar kinetics. **a**, **b** Representative CM images of cells fixed at 12 (**a**) or 9 hpi (**b**), and double-stained for the viral N protein and Rab11 and ERGIC-53 to localize REs/ERC or *cis*-Golgi elements, respectively. The nuclei were visualized by DAPI staining. Both Rab11 and ERGIC-53 display clearly distinct localization patterns in the infected and uninfected (asterisks) cells, due to ERC compaction (**a**) or fragmentation of the Golgi ribbon (**b**). Also note variable positioning of the compact ERCs in IBV-infected cells (**a**, open arrowheads), based on centrosome motility. Besides *cis*-side of the Golgi ribbon, ERGIC-53 also localizes to peripheral IC elements (**b**, white arrowheads). **c**) Quantitation of cells showing ERC compaction (Rab11) or Golgi fragmentation (ERGIC-53 or GM130) in the course of IBV infection. **d**) A merged CM image of cells fixed at 9 hpi and double-stained for Rab11 and the Golgi marker GalNT2 to identify and quantitate cells showing both ERC compaction (open arrowheads) and Golgi fragmentation (white arrowheads). Bars, 5 μm

Staining of uninfected Vero cells with antibodies against Rab11 revealed a predominantly widespread localization pattern. In addition, a “cloud” of Rab11-positive elements was observed next to the nucleus, evidently corresponding to the Golgi-associated pool of REs (Fig. 4a–d; see also Fig. 5a). Strikingly, localization of Rab11 in cells fixed at different times after infection revealed that IBV induces a dramatic reorganization of the endocytic recycling apparatus (Fig. 4). While at 6 hpi the distribution of Rab11-positive REs still resembled that seen in the uninfected cells (Fig. 4a and b), at later timepoints (9 and 12 hpi) the infected cells developed a compact ERC pattern (Fig. 4c–f). Since the widespread Rab11 signal at the same time diminished (compare Fig. 4b and f), this dramatic change is most likely based on MT motor-dependent pericentrosomal accumulation of REs derived from the cell periphery and the fragmenting Golgi ribbon (see below; Saraste and Prydz, 2019; Fujii et al. 2020). Similar results were obtained using antibodies against the TfR (data not shown); however, in this case, the number of cells showing ERC compaction did not increase to the same extent as seen using Rab11 as the marker (Fig. 5c), possibly owing to an effect of IBV infection on the endocytic cycling of this PM receptor.

Previous work has demonstrated that IBV infection also results in Golgi fragmentation, which may promote release of infectious virus (Ruch and Machamer 2011; Westerbeck and Machamer 2015). Therefore, it was of interest to compare the time course of the two virus-induced alterations of the endomembrane system—ERC compaction and Golgi fragmentation—which both were easy to identify (compare uninfected and infected cells in Fig. 5a and b). Besides anti-Rab11, we used antibodies against p58/ERGIC-53 and GM130, two commonly employed markers that localize to *cis*-Golgi cisternae (Saraste et al. 1987; Schweitzer et al. 1988; Nakamura et al. 1995; Saraste and Marie 2018), thus helping to determine the state of the Golgi ribbon (Fig. 5b). Interestingly, the two processes followed very similar kinetics and by 12–14 hpi were detectable in the majority (up to 85%) of the IBV-infected cells (Fig. 5c). It should also be noted that the observed kinetics of Golgi fragmentation and ERC compaction resemble thatof virus release (Fig. 1a), suggesting that these processes are intimately related. Supporting this conclusion, quantitation of cells costained for Rab11 and the *cis*-*medial*-Golgi enzyme GalNAc-transferase 2 (GalNT2) (Fig. 5d) showed that about 80% of cells with ERC compaction also displayed Golgi fragmentation, with the former alteration helping to unambiguously identify the infected cells.

### M protein overlaps with Rab11, but not with LAMP-1

**Fig. 6:**
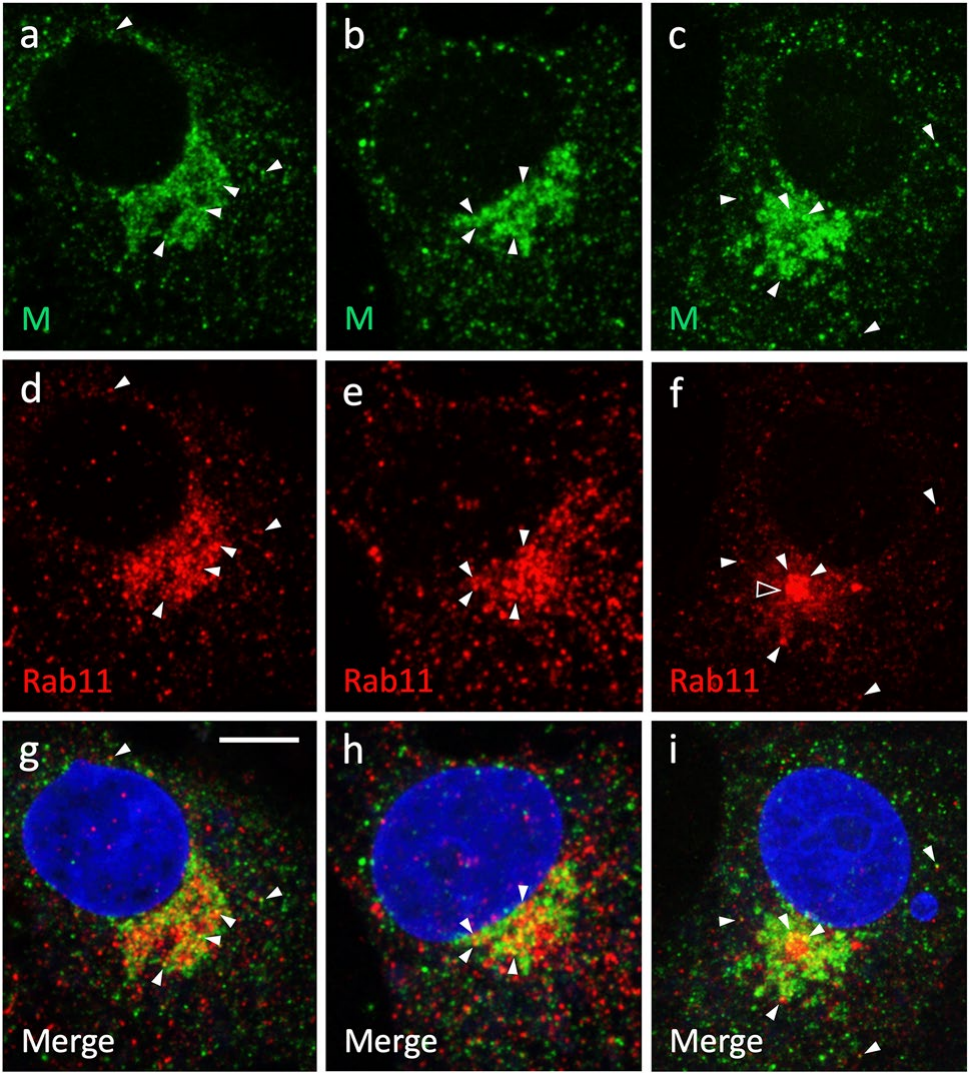
Partial colocalization of the M protein and Rab11 in IBV-infected cells. The cells were fixed at 8 hpi and double-stained with antibodies against the M protein (**a**–**c**) and Rab11 (**d**–**f**) for imaging by CM. The nuclei were visualized by DAPI staining. As indicated by their Rab11 staining patterns, the three cells appear to display variable degrees of ERC compaction (**f**, open arrowhead). Note the codistribution and partial colocalization of the two proteins in the pericentrosomal Golgi region, as well as in some peripheral structures (white arrowheads). Bar, 5 μm

Since the above results suggested that the endocytic recycling system defined by Rab11 plays a role during the late stage(s) of the IBV life cycle, we next examined possible colocalization of the IBV M protein and Rab11 in cells fixed during active virus release at 8 hpi (Fig. 1a). Double-staining of cells displaying different degrees of ERC compaction, and most likely also of Golgi fragmentation (Fig. 5b–d), revealed that the M-protein-containing IC elements and Rab11-positive REs maintain close codistribution in the Golgi region, despite the extensive organelle rearrangements that take place in virus-infected cells. Interestingly, the M protein and Rab11 also showed partial colocalization both in the Golgi area—frequently in the vicinity of the pericentrosomal ERC undergoing compaction—as well in peripheral structures (Fig. 6).

**Fig. 7:**
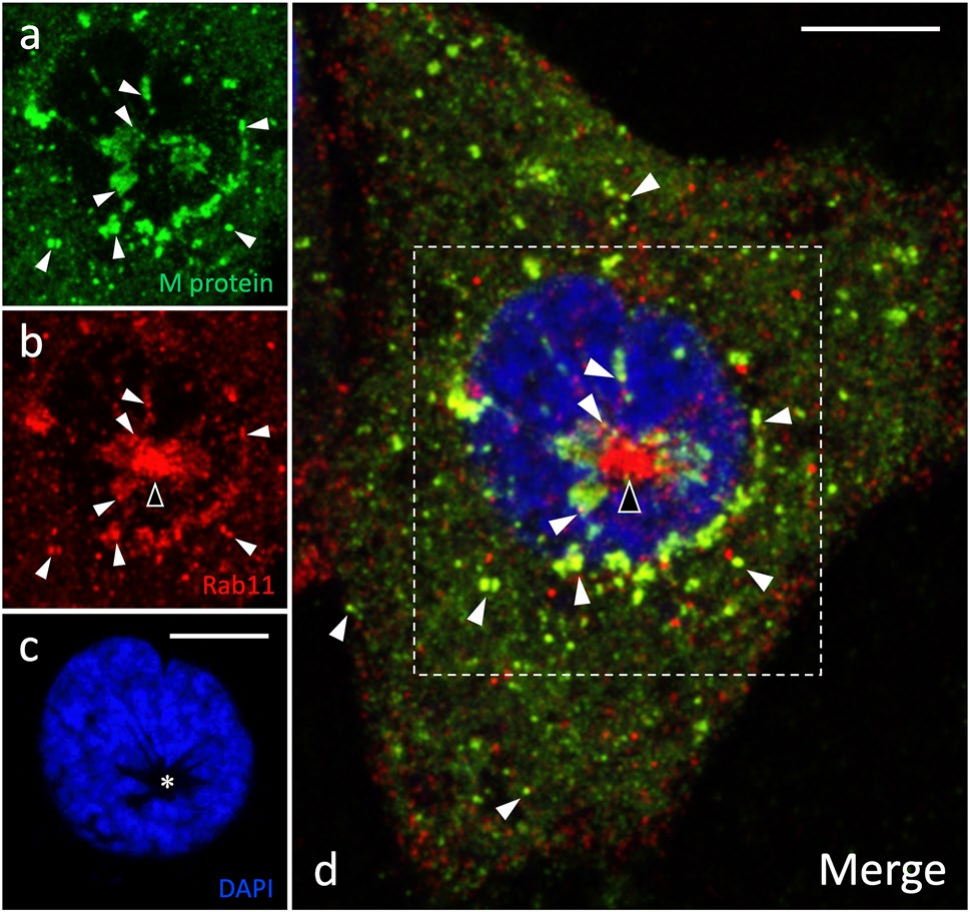
Colocalization of the M protein (**a**) and Rab11 (**b**) during early mitosis. The cells were fixed at 8 hpi and costained for the M protein and Rab11. Nuclear DNA staining by DAPI reveals chromosome condensation (**c**), indicating that the cell has entered prophase. Owing to centrosome motility at prophase, the M-protein-containing IC elements and the Rab11-positive ERC have repositioned underneath the nucleus, forming a distinct nuclear pocket (**c**, asterisk). The two proteins codistribute and partially colocalize both in the pericentrosomal region as well as in punctate structures at the periphery of the mitotic cell (panels** a**, **b** and **d**; white arrowheads). Bars, 5 μm

Besides interphase cells, we compared the localizations of the IBV M protein and Rab11 during mitosis. Namely, our previous studies revealed that during mitotic prophase, when the Golgi ribbon undergoes fragmentation and disassembly (as also occurs during IBV infection), the pericentrosomal IC elements and the ERC—defined by Rab1 and Rab11, respectively—persist and maintain their close connections with each other and the duplicated centrosome that moves to the cell center (Marie et al. 2012; Saraste and Prydz 2019). Notably, imaging of IBV-infected cells during prophase verified that the M-protein-containing IC membranes and the Rab11-positive ERC still remain associated with the centrosome, as it relocates to underneath the nucleus, forming a distinct “nuclear pocket” (Fig. 7). Moreover, partial colocalization of the M protein and Rab11 could be observed in punctate structures close to the compact pericentrosomal ERC, as well as at the cell periphery (Fig. 7). In summary, the above results suggest that a direct functional connection between the virus-containing IC elements and the endocytic recycling apparatus is maintained in the IBV-infected cells.

**Fig. 8:**
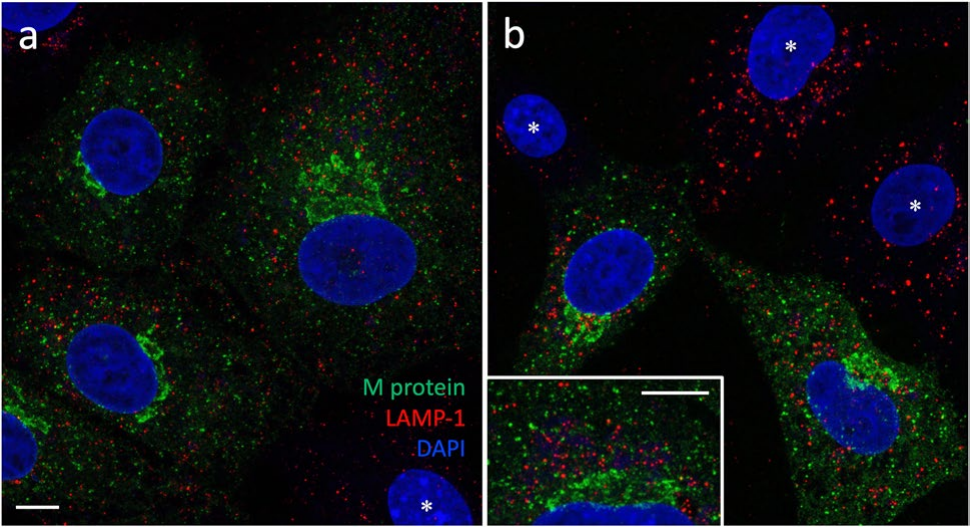
Non-overlapping localizations of the M protein and LAMP-1 in IBV-infected Vero cells. The cells fixed at 8 (**a**) or 12 hpi (**b**) were double-stained for the viral M protein and LAMP-1, and subjected to imaging by CM. Despite their strong expression and joint accumulation in the perinuclear Golgi region in part of the cells (see **b**, inset), the proteins display negligible colocalization. Also note the similar LAMP-1 staining patterns of the infected (M-positive) and uninfected (M-negative; **b**, asterisks) cells. Bars, 5 μm

Finally, because of a recent study providing evidence that β-CoV egress from host cells is based on lysosomal exocytosis (Ghosh et al. 2020), we compared the localizations of the M protein and LAMP-1, a membrane protein predominantly residing in late endosomes and lysosomes. However, double-staining of cells fixed at early or late stages of infection—at 8 or 12 hpi, respectively—revealed negligible colocalization of the two proteins (Fig. 8; see below, Fig. 10), ruling out the possibility that lysosomal secretion plays a major role in IBV release from Vero cells.

**Fig. 9:**
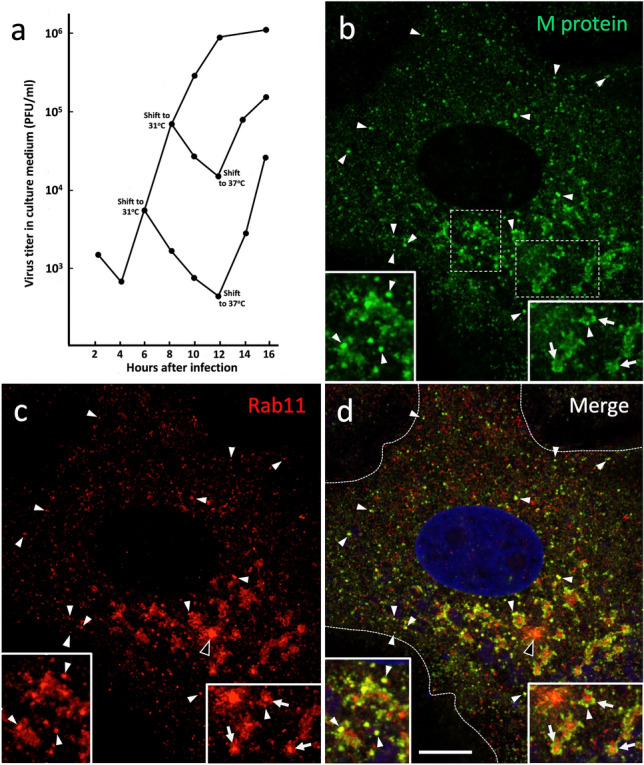
Blocking virus release by low temperature (31 °C) incubation increases the colocalization of the IBV M protein and Rab11 after return of cells to physiological temperature (37 °C). **a** The virus-infected cells grown at 37 °C were shifted at 6 or 8 hpi for various time periods (4–6 h) to 31 °C, followed by return to 37 °C. Medium samples taken at the indicated timepoints were subjected to plaque titration to determine the amounts of infectious virus released. **b**, **c** Double-staining for the M protein and Rab11 of cells shifted at 6 hpi for 2 h to 31 °C, followed by return for 30 min to 37 °C. Despite considerable Golgi fragmentation caused by the low-temperature incubation, the proteins show considerable colocalization in large punctate structures in the Golgi region, highlighted by the insets (arrowheads). Also note the Rab11-positive RE clusters surrounded by M-protein-containing IC elements (right insert, arrows). The ERC is indicated by the open arrowheads in **c** and **d**. Interestingly, the M protein and Rab11 also colocalize in punctate structures of variable size present at the cell periphery (arrowheads), even close to the cell surface (dashed lines in **d**). Bar, 5 μm

**Fig. 10:**
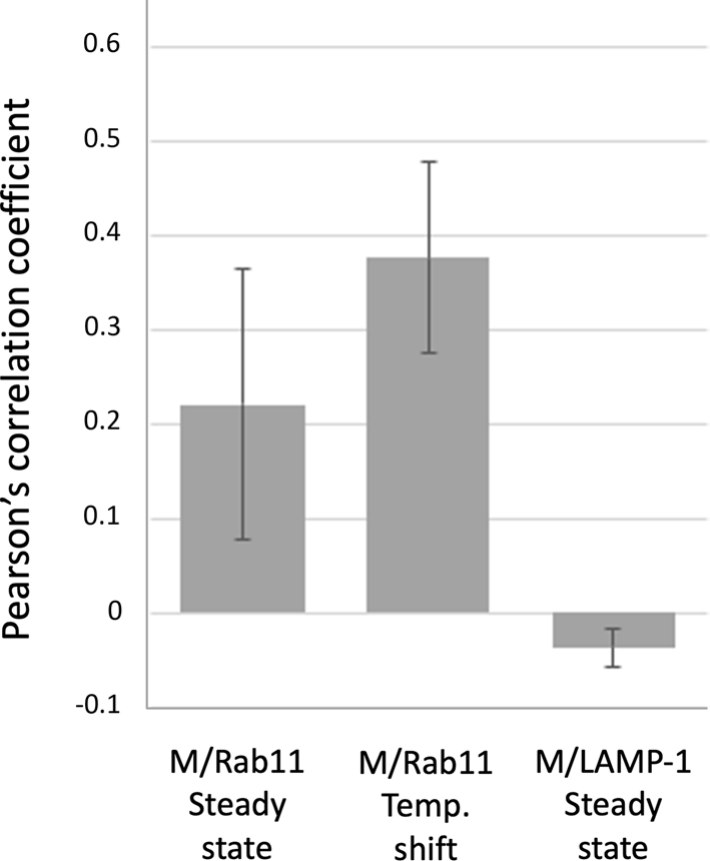
Quantitation of colocalization of the IBV M protein with Rab11 (*n* = 18) or LAMP-1 (*n* = 12) in images of double-stained cells fixed at steady state (at 8 hpi), or following shift of cells at 6 hpi for 2 h to 31 °C and return for 30 min back to 37 °C (*n* = 22), by determination of Pearson’s correlation coefficients (PCCs). See also Supplementary Fig. S1

### Synchronization of virus egress enhances colocalization of M with Rab11

Previous work by Tooze and coworkers using mouse hepatitis virus (MHV)-infected mouse fibroblasts showed that shifting the cells to reduced temperature (31 °C) at 6 or 8 hpi does not significantly affect the intracellular budding of this CoV, but efficiently inhibits its delivery to the extracellular space. However, return of cells back to 37 °C led to rapid MHV release, showing that the low-temperature effect is readily reversible (Tooze et al. 1988). This study motivated us to assess whether similar temperature-shift experiments could be employed to study the egress of IBV from epithelial Vero cells. For this purpose, following the incubation of IBV-infected cells for 6 or 8 h at 37 °C, some of the cultures were shifted for 2–6 h to 31 °C, whereafter some were returned back to physiological temperature (37 °C). Samples were collected from the growth medium at the end of the incubations and subjected to plaque titration to determine the amounts of infectious virus released. Notably, results similar to those reported for MHV (Tooze et al. 1988) were obtained also in the case of IBV (Fig. 9a), encouraging us to apply these temperature manipulations to obtain also better synchronization of the intracellular events leading to IBV release.

Importantly, compared with cells fixed at steady state (Fig. 6), the temperature-shift protocols markedly increased the colocalization of the IBV M protein and Rab11 (Fig. 9b–d), as shown by the quantifications in Fig. 10. However, the extent of colocalization was highly variable between the different cells (Fig. 10), suggesting that the intracellular synchronization achieved correlates with the stage of virus infection. Moreover, CM revealed that incubation at 31 °C, particularly when adopted early during infection, accelerates Golgi fragmentation (Fig. 9b–d). Interestingly, the increased overlap of the M protein and Rab11 affected both large punctate structures, predominantly present in the perinuclear Golgi region, as well as smaller vesicular elements at the cell periphery, close to the PM (Fig. 9b–d). On the basis of their differential localization, it is likely that these punctate Rab11-positive elements of variable size correspond to the large and small CoV-containing carriers described by previous EM studies (Tooze et al. 1987; Ulasli et al. 2012; Ruch and Machamer 2011; Eymieux et al. 2021).

## Discussion

The first characterized function of the endocytic recycling apparatus, in which Rab11 acts as a key regulator, was the retrieval of selected proteins and lipids back to the PM to maintain its compositional and functional properties (Maxfield and McGraw 2004; Grant and Donaldson 2009). Subsequent studies have highlighted novel roles of the REs/ERC in intracellular trafficking: for example, in membrane delivery to phagosomes and autophagosomes (Murray et al. 2005; Husebye et al. 2010; Longatti et al. 2012; Puri et al. 2018), and communication of these compartments with the Golgi apparatus (Wilcke et al. 2000; Taguchi 2013). In fact, it turns out that numerous newly synthesized proteins traverse the endocytic recycling system during their delivery to the PM (Ang et al. 2004; Lock and Stow 2005; Cresawn et al. 2007; Misaki et al. 2010). Since previous studies had revealed that the Rab11-positive post-Golgi REs are functionally connected with the pre-Golgi IC (Marie et al. 2009; Bowen et al. 2017; Saraste and Marie 2018; Kennedy and Hanus 2019), it was tempting to suggest that CoVs make use of such a direct IC–RE link for their cellular exit, instead of taking the conventional secretory route (Saraste and Prydz 2021). The large size of CoVs as secretory cargo, and their significant impact on Golgi organization (Machamer 2013) may also call for their unconventional secretion via the endosomal recycling system, a proposal that has gained experimental support from the present study.

The operation of the REs/ERC at the intersection of endocytic and exocytic transport routes (Grant and Donaldson 2009; Saraste et al. 2009; Taguchi 2013; Saraste and Prydz 2019) may also explain why numerous viruses exploit these compartments during their replication. For example, the ERC is intimately involved in the biogenesis of the assembly compartment of cytomegalovirus, belonging to herpesviruses (Lučin et al. 2018). Regarding RNA viruses, Rab11-dependent pathways have been implicated in the release filoviruses, such as Ebola virus (Nanbo and Ohba 2018), and hepatitis C virus—a flavirus, which buds into the ER lumen (Coller et al. 2012; Bunz et al. 2021). Bunyaviruses, which, like CoVs, assemble by budding at IC/Golgi membranes (Jäntti et al. 1997)—and thus are also likely to share their mode of egress—follow a Rab11-dependent pathway during their transport from the pericentrosomal region to the extracellular space (Rowe et al. 2008). The assembly of retroviruses also starts intracellularly via the association of both viral capsids—consisting of gag proteins and the genomic RNA—and envelope proteins with the ERC, followed by their MT-dependent transport to the PM, where virus assembly by budding is completed (Pereira et al. 2014; Kirschman et al. 2018). Similarly, to support their budding at the PM, ribonucleoproteins (vRNPs) of orthomyxo- (e.g., respiratory syncytial virus) and myxoviruses (e.g., influenza virus)—containing the viral RNA genomes—associate with the ERC membranes to be delivered to the cell surface in RE carriers moving along MT tracks (Amorim et al. 2011; Eisfeld et al. 2011; Bruce et al. 2012).

Obviously, our results raise numerous questions regarding the mechanisms and significance of the ERC compaction taking place in IBV-infected cells. However, recent studies of influenza virus may provide some answers. Namely, Kawaguchi and coworkers discovered that viral infection of HeLa cells induces Rab11 activation and increases MT nucleation, owing to cell-cycle-independent maturation of the centrosome (Kawaguchi et al. 2015). As a consequence, cholesterol-rich REs accumulate at the pericentrosomal ERC, giving rise to a similar compaction of this compartment as described in the present study. In addition, the authors provided evidence for the formation of cholesterol-rich domains (“lipid rafts”) within the ERC that, by triggering membrane association of the vRNPs, initiate virus assembly (Kawaguchi et al. 2015). Interestingly, it has been recently reported that similar formation of cholesterol-rich nanodomains around the excessively acylated spike (S) protein of SARS-CoV-2 drives virus budding at IC membranes, which normally display a low cholesterol content (Mesquita et al. 2021). Therefore, it is possible that compaction of the ERC, by maintaining an intimate connection between the REs and the expanding IC after Golgi disassembly, is required not only for virus egress, but also to boost lipid exchange between these compartments to support virus budding at specific IC subdomains.

In conclusion, our results provide the first evidence that CoVs—like many other enveloped viruses, budding either intracellularly or at the cell surface—employ the endocytic recycling compartments defined by Rab11 to gain exit from their host cells. Indeed, by harnessing the functional connection between the IC elements and REs at the cell center, influenza viruses and CoVs may have adopted similar replication strategies without sharing the same site of assembly. However, since the present and previous studies provide conflicting data on the role of the endocytic and secretory compartments in CoV egress (Tooze et al. 1987; Ulasli et al. 2012; Ghosh et al. 2020; Bracquemond and Muriaux 2021; Eymieux et al. 2021; Prydz and Saraste 2022), future work employing various EM techniques and addressing the effects of organelle inhibitors, such as Brefeldin A (Ghosh et al. 2020), on CoV release is required to settle this issue. For the moment, the possibility cannot be ruled out that different CoVs favor alternative routes depending on the host cell or tissue.

## Supplementary Information

Below is the link to the electronic supplementary material.

Supplementary file1 (TIF 20180 KB) Examples of images of cells double-stained for the IBV M protein and Rab11 or LAMP-1 and subjected to colocalization analysis. The original images, images after reduction of background fluorescence at both the green and red channels (threshold level 10), as well as the corresponding scatter plots and PCC values are shown. See also Fig. 10.

## Data Availability

All data generated or analyzed during this study are included in this published article (and its supplementary information files).
