# Supplementary figures and images for: Evidence for the role of Rab11-positive recycling endosomes as intermediates in coronavirus egress from epithelial cells
Source: Histochem Cell Biol. 2022 May 23;158(3):241–51. doi: 10.1007/s00418-022-02115-y (PMC9124743; doi:10.1007/s00418-022-02115-y)

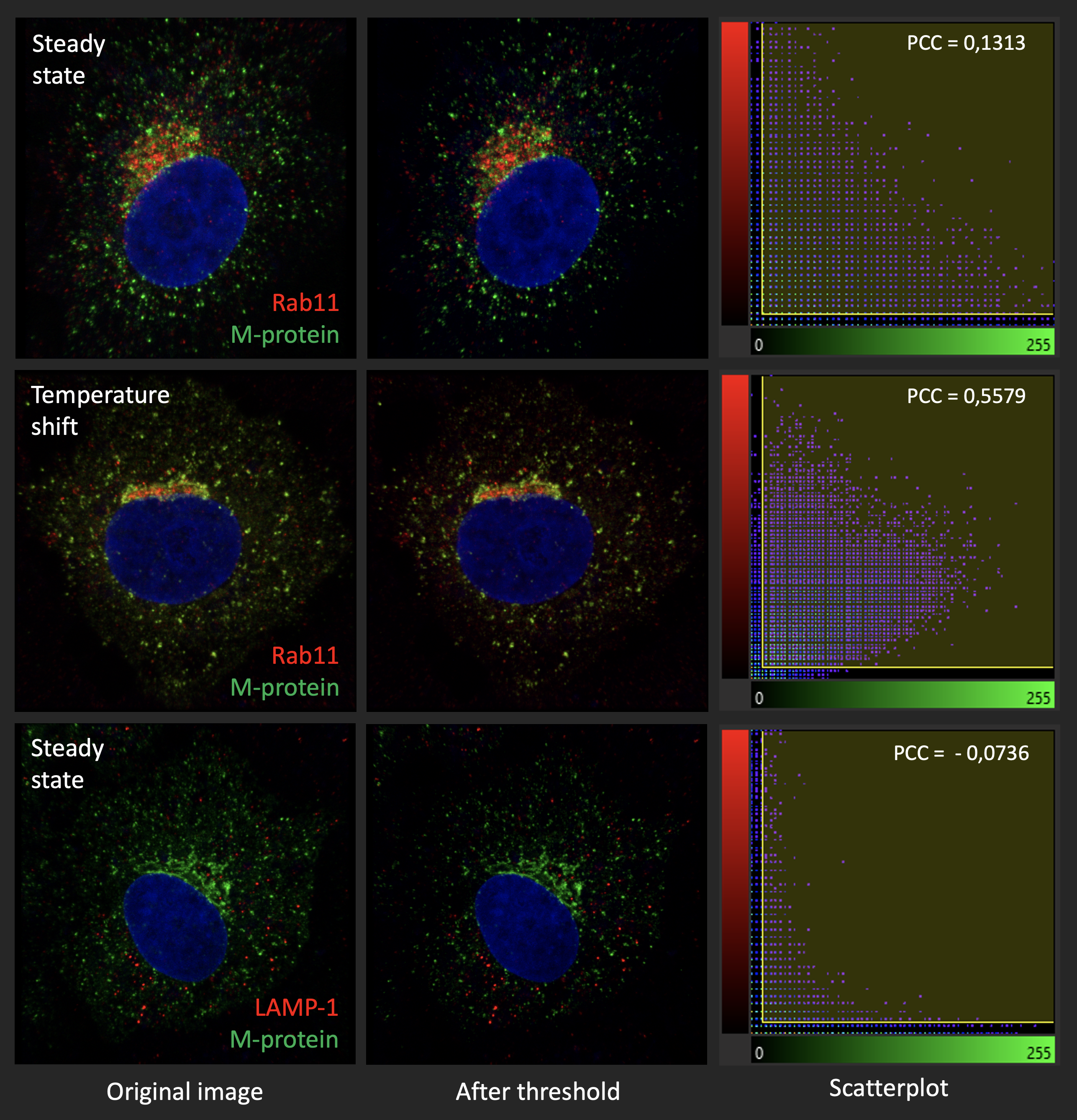

Supplement: Supplementary file 1 — Supplementary file1 (TIF 20180 KB) Examples of images of cells double-stained for the IBV M protein and Rab11 or LAMP-1 and subjected to colocalization analysis. The original images, images after reduction of background fluorescence at both the green and red channels (threshold level 10), as well as the corresponding scatter plots and PCC values are shown. See also Fig. 10. [file 418_2022_2115_MOESM1_ESM.tif]
